# The CXCL5/CXCR2 axis contributes to the epithelial-mesenchymal transition of nasopharyngeal carcinoma cells by activating ERK/GSK-3β/snail signalling

**DOI:** 10.1186/s13046-018-0722-6

**Published:** 2018-04-17

**Authors:** Wen-Ze Qiu, Hai-Bo Zhang, Wei-Xiong Xia, Liang-Ru Ke, Jing Yang, Ya-Hui Yu, Hu Liang, Xin-Jun Huang, Guo-Ying Liu, Wang-Zhong Li, Yan-Qun Xiang, Tie-Bang Kang, Xiang Guo, Xing Lv

**Affiliations:** 1 Department of Nasopharyngeal Carcinoma, Sun Yat-sen University Cancer Center, 651 Dongfeng East Road, Guangzhou, 510060 People’s Republic of China; 2 Collaborative Innovation Center for Cancer Medicine, State Key Laboratory of Oncology in South China, Sun Yat-sen University Cancer Center, 651 Dongfeng East Road, Guangzhou, 510060 People’s Republic of China; 3 Department of Radiation Oncology, Shandong Cancer Hospital Affiliated to Shandong University, Shandong Academy of Medical Sciences, Jinan, Shandong China; 4 Department of Radiation Oncology, Shanghai Proton and Heavy Ion Center, 4365 Kangxin Road, Shanghai, 201321 People’s Republic of China

**Keywords:** CXCL5, CXCR2, Epithelial-to-mesenchymal transition, Distant metastasis, Nasopharyngeal carcinoma

## Abstract

**Background:**

Distant metastasis is the major cause of treatment failure in patients with nasopharyngeal carcinoma (NPC). Although several biomarkers correlate with metastasis and prognosis, the molecular mechanisms of NPC development and progression remain unclear.

**Methods:**

Quantitative RT-PCR (qRT-PCR), western blotting, cell growth, foci formation, migration and invasion assays, and xenograft mouse models were utilized to examine the expression levels and functions of the CXCL5/CXCR2 axis in NPC. A luciferase reporter assay, western blotting, immunofluorescence, and migration and invasion assays were used to identify and verify the ERK/GSK-3β/Snail signalling pathway.

**Results:**

CXCL5 was significantly increased in the sera of NPC patients, and high expression levels of CXCL5/CXCR2 in NPC primary tissues indicated poor survival. CXCL5 and CXCR2 were upregulated in NPC cell lines. Ectopic expression of the CXCL5/CXCR2 axis promoted NPC cell migration and invasion in vitro and the formation of lung metastases in vivo. Mechanistically, the dual overexpression of CXCL5 and CXCR2 promoted cell spreading by inducing the epithelial-mesenchymal transition (EMT) through the activation of the ERK/GSK-3β/Snail signalling pathway.

**Conclusion:**

The CXCL5/CXCR2 axis contributes to the EMT of NPC cells by activating ERK/GSK-3β/Snail signalling, and this axis may be a potential diagnostic marker and therapeutic target for patients with NPC.

**Electronic supplementary material:**

The online version of this article (10.1186/s13046-018-0722-6) contains supplementary material, which is available to authorized users.

## Background

Nasopharyngeal carcinoma (NPC) is one of the most prevalent tumour types in Southeast Asia and southern China with a reported incidence of 15–50 per 100,000 people [1–3]. With the advances in radiotherapy and chemotherapy, patient outcomes have significantly improved; however, the rate of treatment failure is high due to local recurrence and distant metastasis, which are the major contributors to NPC-related mortality [4–6]. Therefore, finding new therapeutic targets by further elucidating the molecular mechanisms of NPC metastasis is necessary and urgent.

The epithelial-mesenchymal transition (EMT) plays an essential role in tumour invasion and metastasis [7, 8]. This transition is characterized by a loss of epithelial markers, including E-cadherin, and the acquisition of new mesenchymal proteins, such as Vimentin [9]. Key regulators of the EMT, including ZEB1, ZEB2, Slug, Snail and Twist, are potential targets for the prevention of cancer metastasis [10, 11].

As a constitutively active serine/threonine kinase, glycogen synthase kinase-3 beta (GSK-3β) is active in resting epithelial cells [12] and can be inactivated by various signalling mechanisms, including the Wnt [13], extracellular signal-regulated kinase (ERK)1/2 MAPK [14], and lipid kinase phosphatidylinositol-4,5-bisphosphate 3-kinase (PI3K)/AKT [15] pathways. Previous studies have reported that GSK-3β can maintain epithelial properties by binding to Snail and promoting its proteasomal degradation [16].

Epithelial neutrophil-activating peptide-78 (CXCL5) is a member of the CXC chemokine family and is upregulated in pancreatic [17], prostate [18], and bladder [19] cancers and in hepatocellular carcinomas [20], and the overexpression of CXCL5 is associated with advanced tumour stage, local invasion and metastatic potential. Previously, we demonstrated that a high serum CXCL5 level is associated with advanced lymph node classification, distant metastasis and tumour progression in NPC patients [21]. The overexpression of the CXCL5-specific receptor, CXCR2, has been verified in a variety of tumour cells, including breast [22], lung [23] and colon cancer cells [24], and all cancers with a high metastatic index. The CXCL5/CXCR2 axis can promote the EMT of HCC cells through the activation of the PI3K/Akt/GSK-3β/Snail signalling pathway [20]. Additionally, the CXCL5/CXCR2 axis has also been found to enhance human colorectal cancer metastasis through the activation of the ERK/Elk-1/Snail and AKT/GSK3β/β-catenin pathways [25]. However, the underlying mechanism by which the CXCL5/CXCR2 axis functions in mediating the migration and invasion of NPC cells remains largely unclear. Here, we explored the possible clinical and biological roles of the CXCL5/CXCR2 axis in NPC. Investigation of the mechanisms demonstrated that the CXCL5/CXCR2 axis promotes metastasis of NPCs by inducing the EMT through the activation of the ERK/GSK-3β/Snail signalling pathway.

## Methods

### Cell culture

NP69 immortalized nasopharyngeal epithelial cells were maintained in keratinocyte/serum-free medium (Invitrogen, Grand Island, NY, USA). Four human NPC cell lines (S18, S26, 5-8F and 6-10B) were cultured in Dulbecco’s modified Eagle’s medium (DMEM; Invitrogen, Carlsbad, CA, USA) supplemented with 10% foetal bovine serum (FBS; Gibco, Grand Island, NY, USA). S18 and 5-8F are considered to be highly metastatic cell lines, whereas S26 and 6-10B are considered poorly metastatic cell lines according to previous studies [26, 27].

### Patients and tissue specimens

Prior written patient consent and Institutional Research Ethics Committee approval were obtained. No patients received treatment prior to biopsy.

Fifty NPC specimens and fifty chronic nasopharyngitis samples were obtained from patients who underwent nasopharyngeal biopsies before treatment at Sun Yat-sen University Cancer Center (SYSUCC) between November of 2009 and December of 2009.

Additionally, paraffin-embedded NPC specimens were obtained from 107 patients with histologically and clinically diagnosed non-metastatic NPCs who were treated at SYSUCC between August of 1999 and March of 2000 (Table 1). The tumour stages were classified according to the sixth edition of the Union for International Cancer Control (UICC) staging system. All 107 patients received radical radiotherapy. The median follow-up duration was 116 months (range, 8–139 months) for the entire cohort. Thirty-seven patients (34.6%) were lost to follow-up, including 15 with locoregional relapse, 20 with distant metastasis and two with both. In total, 44 (41.1%) patients died, including 36 cancer-specific deaths and 8 non-cancer-specific deaths.

**Table 1:** Clinicopathological correlation of CXCL5 expression and CXCR2 expression in NPC

	All patients	CXCL5			CXCR2		
	*n* = 107(%)	High (*n* = 44)	Low (*n* = 63)	*P*	High (*n* = 52)	Low (*n* = 55)	*P*
Age (year)							
< 50	68(63.6)	27	41	0.694	30	38	0.221
≥ 50	39(36.4)	17	22		22	17	
Gender							
Male	84(78.5)	35	49	0.827	37	47	0.072
Female	23(21.5)	9	14		15	8	
Tumor classification							
T1–2	59(55.1)	22	37	0.372	27	32	0.515
T3–4	48(44.9)	22	26		25	23	
Nodal classification							
N0–1	78(72.9)	26	52	***0.007***	33	45	***0.033***
N2–3	29(27.1)	18	11		19	10	
Clinical classification							
I-II	44(41.1)	14	30	0.102	20	24	0.587
III-IVb	63(58.9)	30	33		32	31	
Local-regional recurrence							
No	90(84.1)	35	55	0.280	46	44	0.231
Yes	17(15.9)	9	8		6	11	
Distant metastasis							
No	85(79.4)	32	53	0.055	37	48	***0.039***
Yes	22(20.6)	12	10		15	7	
Progression							
No	70(65.4)	24	46	***0.048***	32	38	0.412
Yes	37(34.6)	20	17		20	17	

Progression is defined as local-regional recurrence and/or distant metastasis after initial treatment. Statistical significance (*P* < 0.05) is shown in bold and italic

### Enzyme-linked immunosorbent assay (ELISA)

The serum CXCL5 levels of the 100 aforementioned patients (50 patients with NPCs and 50 patients with chronic nasopharyngitis) were measured with a commercially available ELISA kit (Quantikine Human ENA-78; R & D Systems, Minneapolis, MN, USA) in accordance with the manufacturer’s instructions. The secretions of CXCL5 into the media collected from cultures of the NPC or NP69 cells were also determined by ELISA. The ELISA was performed as described previously [28, 29].

### Immunohistochemical staining (IHC) and histologic evaluation

Paraffin-embedded blocks were sectioned for IHC staining as performed previously [30]. Briefly, paraffin-embedded tissue specimens were deparaffinized and rehydrated. Antigenic retrieval was performed with citrate buffer (pH 6.0) using a 700-W microwave for 10 min. The sections were then incubated in H_2_O_2_ (3%) for 10 min, blocked in goat serum at room temperature for 30 min and incubated with anti-CXCL5 (1:100) or anti-CXCR2 (1:200) antibodies overnight at 4 °C. An EnVision kit (DAKO, Carpinteria, CA, USA) was used to detect the primary antibodies followed by 3, 3-diaminobenzidine substrate visualization and counterstaining with haematoxylin. The intensity of IHC staining in the tumour cells was scored independently by two pathologists using the semiquantitative immunoreactive score (IRS) scale (for staining intensity: no staining = 0; weak staining =1; moderate staining = 2; and strong staining = 3. For the percentage of stained cells, 0% = 0; 1%–10% = 1; 11%–50% = 2; 51%–80% = 3; and 81%–100% = 4). The staining score for each tissue was calculated by multiplying the staining value by the percentage category value, and the average of the scores from the two referees was used as the final score. The median and range of the IRS for each antibody and the definitions of high-expression and low-expression of CXCL5 and CXCR2 used in this study are listed in Additional file 1: Table S1.

### Plasmids

The psi-LVRU6GP-shRNA-CXCR2, psi-LVRU6GP-shRNA-CXCL5, pEZ-Lv105-CXCR2 and pEZ-Lv105-CXCL5 lentiviral vectors were purchased from GeneCopoeia, Inc., and the target short hairpin RNA (shRNA) sequences are listed in Additional file 2: Table S2. The psi-LVRU6GP and pReceiver-Lv105 lentiviral vectors were used as controls. A small interfering RNA (siRNA) was used to knock down Snail and was constructed and synthesized by Guangzhou RiboBio Co., Ltd. (Guangzhou, China); the target sequences are listed in Additional file 2: Table S2. The pcDNA3.1-GSK-3βS9A plasmids were purchased from Shanghai GeneChem Co., and pcDNA3.1 plasmids were used as a control. All transfections have been described previously [31].

### Western blot analysis and immunofluorescence assay

The western blotting and immunofluorescence assays were performed as previously described [32–34]. The primary antibodies and dilutions are listed in Additional file 3: Table S3. The grey values of the target bands were quantified with ImageJ software.

### qRT-PCR

qRT-PCR was performed using a LightCycler 480 instrument (Roche Diagnostics) and SYBR Premix Ex Taq (TaKaRa) according to the manufacturer’s instructions. To quantify the cancer metastases in the mouse lungs, specific primers for human HPRT that do not cross-react with its mouse counterpart were designed as described [35–37]. The primers used are listed in Additional file 2: Table S2.

### Cell growth and foci formation assays

For cell growth assay, cells were seeded in 96-well plate at a density of 1 × 10^3^ per well and cell growth rate was assessed by Cell Counting Kit-8 (Dojindo). Cellular growth curves were plotted by using the cellular viability values. For foci formation assay, 1 × 10^3^ cells per well were seeded dispersedly in 6-well plate. After one-week culture, cell colonies were counted by crystal violet staining. The results are expressed as mean ± SD of three independent experiments.

### Migration and invasion assays

Migration and invasion assays were used to evaluate the migration and invasion abilities of cells. Transwell assays were performed using a Boyden chamber containing 24-well transwell plates (BD Inc.) with 8-μm membrane pores. All experiments were performed in duplicate and repeated three times. For the Transwell migration assay, 2 × 10^4^ (S18 and 5-8F) or 4 × 10^4^ (S26 and 6-10B) cells in 400 μl of serum-free DMEM were added to the cell culture inserts without extracellular matrix coating. DMEM containing 10% FBS was added to the bottom chamber. After approximately 18 h of incubation, the cells on the lower surface of the filter were fixed, stained, and examined using a microscope. For the Transwell invasion assay, the membrane was coated with 50 μl of 1:5 diluted Matrigel (BD Biosciences). After the Matrigel had solidified at 37 °C overnight, 4 × 10^4^ (S18 and 5-8F) or 1 × 10^5^ (S26 and 6-10B) cells in 400 μl of serum-free DMEM were added to the cell culture inserts, while the lower chamber was filled with DMEM containing 10% FBS. The Boyden chamber was then incubated at 37 °C with 5% CO_2_ for approximately 24 h. The subsequent staining and observation procedures were the same as for the migration assays.

### Luciferase reporter assay

The indicated stable cells were seeded in Cignal Finder 10-Pathway Reporter Array plates (QIAGEN, Dusseldorf, GER) when the cell density and cell viability met the transfection conditions. The protocol of the Luciferase Cignal Finder Reporter Array Plate Format Handbook was followed for developing the assays. A Dual-Glo Luciferase Assay System (Promega, Madison, WI) was used to measured dual-luciferase signals after reverse transfection. Three independent experiments were performed, and the calculated means and standard deviations are presented.

### Annexin V/Propidium Idodide (PI) double-staining assay

Annexin V/PI staining was performed using the Annexin V-fluorescein isothiocyanate apoptosis detection kit. Cells (3.0 × 10^5^ per mL) were seeded into six-well plate with 2 mL in each well, then treated with 50 nM (the same concentration used in the migration assay) of trametinib for 24 h. Both floating and attached cells were collected, washed with ice-cold phosphate buffered saline (PBS) twice, then incubated at room temperature in the presence of media binding reagent and Annexin V-FITC for 15 min in the dark. After washing with PBS, the cells were resuspended in ice-cold 1 × binding buffer and treated with 10 μL propidium iodide (30 μg/ml) on ice in the dark. Apoptosis was quantified by flow cytometry (Becton Dickinson) at the wavelength of 488 nm immediately and analyzed by the Cell-Quest software.

### Animal experiments

All animal experiments were performed in accordance with protocols approved by the Research Animal Resource Center of Sun Yat-sen University. Animals were obtained from Beijing Vital River Laboratory Animal Technology Co., Ltd. (Beijing, China). A lung metastasis model was used [35]. Briefly, the indicated stable cells were harvested and washed twice with PBS. Approximately 1 × 10^6^ cells resuspended in 150 μl of PBS were injected into the tail veins of 4-week-old male athymic mice. All mice were sacrificed at 7 weeks after injection. The lungs of the mice were weighed, and the number of metastatic nodules in the lungs was counted. The harvested lungs were fixed in formalin (4 μmol/L). Haematoxylin and eosin (H & E) staining was performed for histologic assessment. Total RNA was extracted for qRT-PCR analysis of hHPRT mRNA expression.

### Statistical analysis

Statistical analyses were performed using SPSS 19.0 (IBM, Chicago, IL, USA). Quantitative data between groups were compared using Student’s *t*-test. Categorical data were analysed by a chi-square test or Fisher’s exact test. Actuarial rates of survival were calculated using the Kaplan-Meier method, and differences were compared using log-rank tests. Multivariate analysis was performed using an adjusted Cox proportional hazards model. A 2-tailed *P* value less than 0.05 was considered significant.

## Results

### CXCL5 and CXCR2 are upregulated in NPC tissues and highly metastatic NPC cell lines, and CXCL5 is significantly increased in the sera of NPC patients

**Fig. 1:**
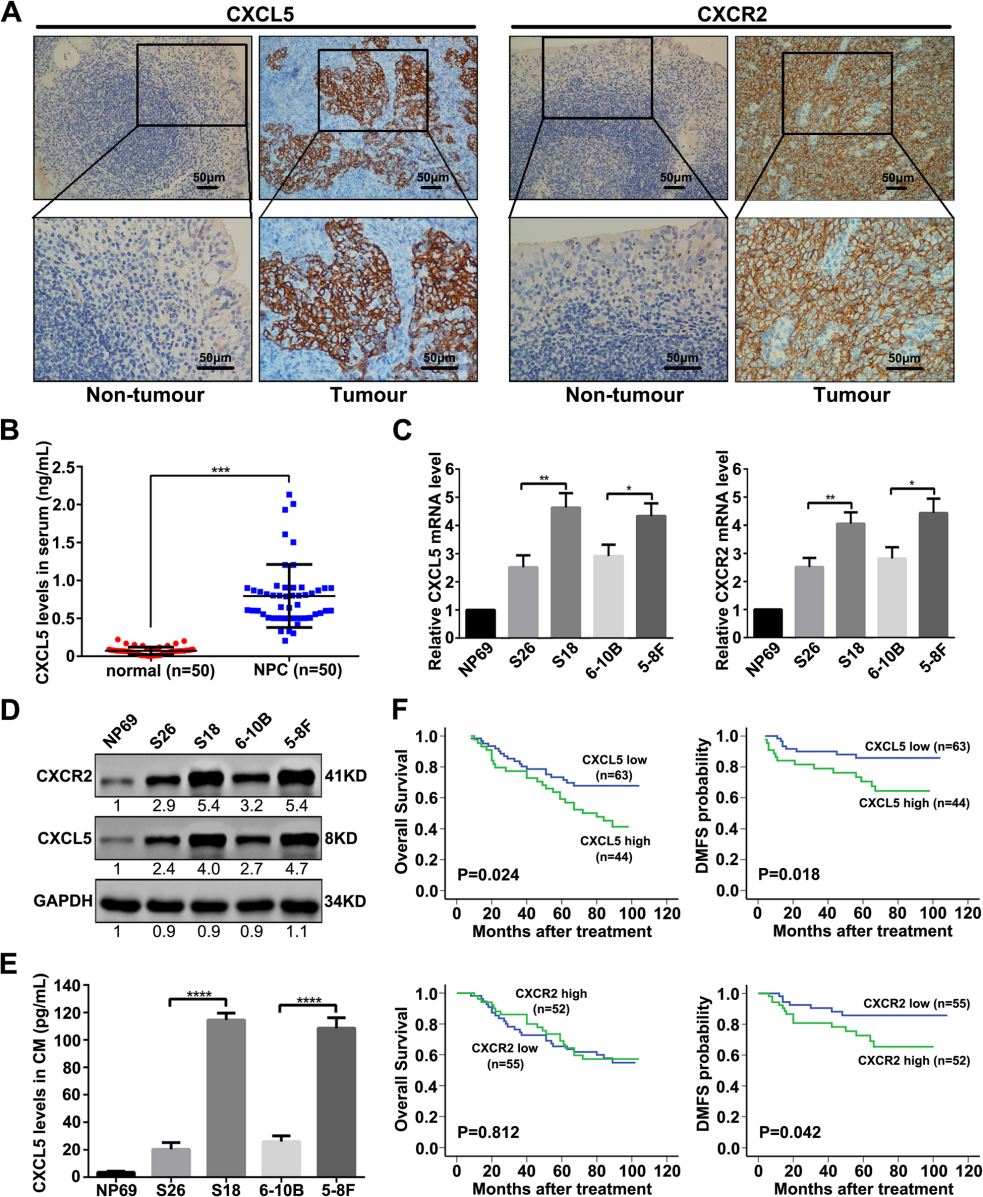
CXCL5 and CXCR2 are upregulated in NPC cell lines and tissue samples. **a** Representative images of CXCL5 expression and CXCR2 expression in non-tumour tissue and NPC tumour tissue detected by IHC. **b** The results of ELISAs of the human sera showed that the mean serum CXCL5 concentration of 50 NPC patients was significantly higher than that of 50 non-tumour patients. ***P < 0.001. **c**, **d** Upregulations of CXCL5 and CXCR2 were observed in different NPC cell lines compared with non-tumour control cells based on qRT-PCR and western blotting. NP69 cells were used as controls. *P < 0.05, **P < 0.01. **e** Higher levels of CXCL5 in the conditioned media (CM) were observed in the different NPC cell lines compared with the NP69 cells using ELISA. ****P < 0.0001. **f** Kaplan-Meier analysis indicated that higher CXCL5 expression was significantly associated with poorer OS (*P* = 0.024) and lower DMFS (*P* = 0.018), and higher CXCR2 expression was significantly associated with lower DMFS (*P* = 0.042), but not with poorer OS (*P* = 0.812)

The expression of both CXCL5 and CXCR2 was higher in the NPC primary tissues than in the non-tumour tissues at the protein level as quantified by IHC, and the expression of CXCL5 was primarily localized to the tumour cells rather than the mesenchymal portion of the cancerous tissue (Fig. 1a). ELISA analyses also revealed that the serum CXCL5 level was significantly higher in NPC patients than in the non-tumour patients (*P <* 0.001) (Fig. 1b). Next, we found that CXCL5 and CXCR2 were overexpressed in the NPC cell lines compared with the immortalized nasopharyngeal epithelial cell line NP69. The overexpression of CXCL5 and CXCR2 in the highly metastatic cell lines (S18 and 5-8F) was much more obvious than that in the poorly metastatic cell lines (S26 and 6-10B) (Fig. 1c and d). The levels of CXCL5 in the supernatants of the NP69 and NPC cell lines as detected by ELISA were consistent with the results from the qRT-PCR and western blotting (Fig. 1e).

### Clinical significance of the high expression levels of CXCL5 and CXCR2 in NPC

As shown in Table 1, a high expression level of CXCL5 in the NPC primary tissues was significantly associated with nodal classification and progression, and a high expression level of CXCR2 was significantly associated with nodal classification and distant metastasis. As shown in Fig. 1f, a higher level of CXCL5 expression was significantly correlated with poorer overall survival (OS) and distant metastasis-free survival (DMFS), and a higher level of CXCR2 expression was significantly correlated with lower DMFS but not with poorer OS.

Age, gender, histological type, and TNM classification have been demonstrated to influence local-regional recurrence and distant metastasis in NPC patients [38–40]. Thus, in our study, a Cox multivariate analyses that included age, gender, WHO histological type, tumour classification, nodal classification, clinical classification, and CXCL5 and CXCR2 statuses was performed, and the results suggested that a high level of CXCL5 was an independent, unfavourable prognostic indicator for OS and DMFS (Table 2).

**Table 2:** Cox proportional hazard regression analyses for 10-year OS and 10-year DMFS

Prognosis	Wald	*P*	Exp(B)	95% CI for Exp(B)	
				Lower	Upper
OS					
Age(year) ≥50 vs.<50	1.577	0.215	1.643	0.788	3.319
Gender Female vs. Male	3.298	0.081	2.506	0.907	6.387
Histological type U vs. D	0.441	0.526	0.745	0.253	2.479
T classification T2–4 vs. T1	9.309	0.003^a^	3.568	1.521	6.517
N classification N1-3vs. N0	12.505	0.001^a^	2.389	1.543	4.228
Clinical classification II-IVb vs. I	6.218	0.014^a^	0.487	0.119	0.668
CXCL5 expression High vs. Low	4.885	0.027	1.960	1.079	3.560
CXCR2 expression High vs. Low	1.295	0.255	0.691	0.365	1.307
DMFS					
Age(year) ≥50 vs.<50	1.267	0.234	2.137	0.802	4.304
Gender Female vs. Male	3.137	0.091	3.267	0.798	13.223
Histological type U vs. D	0.702	0.564	0.523	0.158	2.694
T classification T2–4 vs. T1	3.236	0.079^a^	2.413	0.903	6.446
N classification N1-3vs. N0	6.533	0.004^a^	2.578	1.131	6.327
Clinical classification II-IVb vs. I	2.786	0.095^a^	0.413	0.251	1.099
CXCL5 expression High vs. Low	5.169	0.023	2.740	1.149	6.532
CXCR2 expression High vs. Low	1.700	0.192	1.879	0.728	4.849

^a^ adjusted *P*-values < 0.017 were considered statistically significant

*OS* overall survival, *DMFS* distant metastasis-free survival, *sCXCL5* serum CXCL5, *D* differentiated non-keratinized carcinoma, *U* undifferentiated non-keratinized carcinoma, *CI* confidence interval

### Overexpression of the CXCL5/CXCR2 axis promotes NPC cell migration and invasion in vitro and increases lung metastasis in vivo

**Fig. 2:**
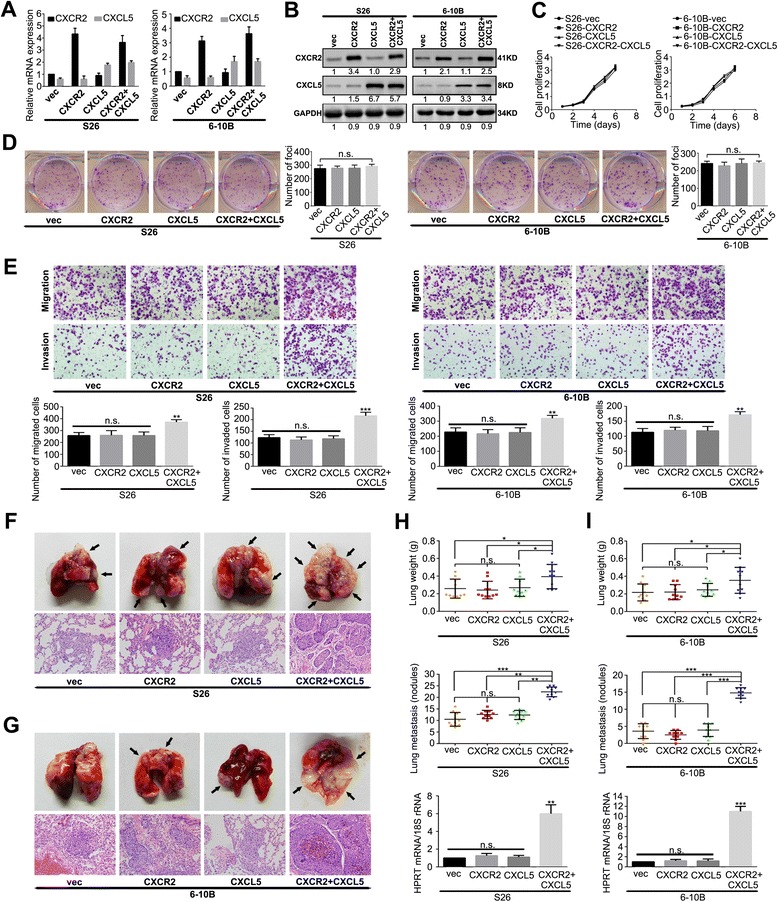
Overexpression of CXCL5/CXCR2 axis promotes migration and invasion in vitro and lung metastasis in vivo. **a** The relative mRNA levels of CXCL5/CXCR2 in the indicated stable cell lines were determined by qRT-PCR. Mean ± SD of triplicate samples are shown; n = 3. **b** Relatively high expression of CXCL5 and CXCR2 were confirmed by western blotting in the CXCL5/CXCR2-overexpressing S26 and 6-10B cells compared with the vector control cells. **c** The cell growth rates were compared between the CXCR2-, CXCL5-, CXCR2 + CXCL5- and empty vector-transfected cells using cell growth assays. **d** Representative images and summaries of the foci formation induced by CXCR2- and CXCL5-transfected cells and vector control cells. **e** The cell migration and invasion of the indicated stable cell lines were determined as described in the Materials and Methods. Representative images of the migrated and invaded cells are shown. **f**, **g** The indicated stable transfected cells were injected into the lateral tail veins of nude mice. The superior and inferior panels present the macroscopic appearance of metastatic lung tumours and H & E staining, respectively. The arrows indicate the metastatic nodules. Original magnification: 20 × objective. **h**, **i** The tumour weights and numbers of metastases per lung in the mice that were injected with the indicated stable cells and the expression of human HPRT mRNA relative to mouse 18S rRNA in the lungs of the tumour-bearing nude mice are shown in H and I, respectively. There were 10 mice in each group. These experiments were repeated at least three times. Error bars, mean ± SD. *P < 0.05, **P < 0.01, ***P < 0.001

The S26 and 6-10B cell lines were transfected with CXCL5 and/or CXCR2 expression plasmids to upregulate the expression of CXCL5 and/or CXCR2. Cells transfected with blank vector were used as the control (vec). qRT-PCR and western blotting were conducted to examine the mRNA and protein levels of CXCL5 and/or CXCR2 in the S26 and 6-10B stable cell lines. As shown in Fig. 2a and b, transfection with the CXCL5 and/or CXCR2 expression plasmids caused a significant increase in the mRNA and protein expression of CXCL5 and/or CXCR2 in the S26 and 6-10B stable cell lines when compared with the vec group. ELISA also revealed significantly increased levels of CXCL5 protein in the stable CXCL5 overexpressing cell lines (Additional file 4: Figure S1). The results of the cell growth and foci formation assays revealed that the overexpression of CXCL5 and/or CXCR2 did not influence NPC cell growth, tumour formation or cell proliferation (Fig. 2c and d). However, we found that the cells expressing high levels of CXCL5/CXCR2 together exhibited greater migration and invasion potentials than the other stable cell lines (Fig. 2e).

Next, we developed an in vivo lung metastasis model by injecting S26 or 6-10B cells and their derived cells into the lateral tail veins of nude mice for 7 weeks. As assessed by H & E staining (Fig. 2f and g), overexpression of the CXCL5/CXCR2 axis resulted in significant increases in lung weight and the number of metastatic pulmonary nodules (Fig. 2h and i). Additionally, the individual tumour burden per lung was measured by qRT-PCR using primers specific for the human housekeeping gene *HPRT*, which detects human but not mouse HPRT mRNA. The expression level of human HPRT in the lungs that had been infiltrated with NPC cells expressing high levels of CXCL5/CXCR2 together were higher than those in the lungs that had been infiltrated with cells expressing high levels of CXCL5 or CXCR2 alone and low levels of CXCL5/CXCR2 together (Fig. 2h and i). Collectively, these results indicated that the overexpression of the CXCL5/CXCR2 axis increases distant metastasis of NPCs in a mouse model.

### Silencing the CXCL5/CXCR2 axis inhibits NPC cell migration and invasion in vitro and decreases lung metastasis in vivo

**Fig. 3:**
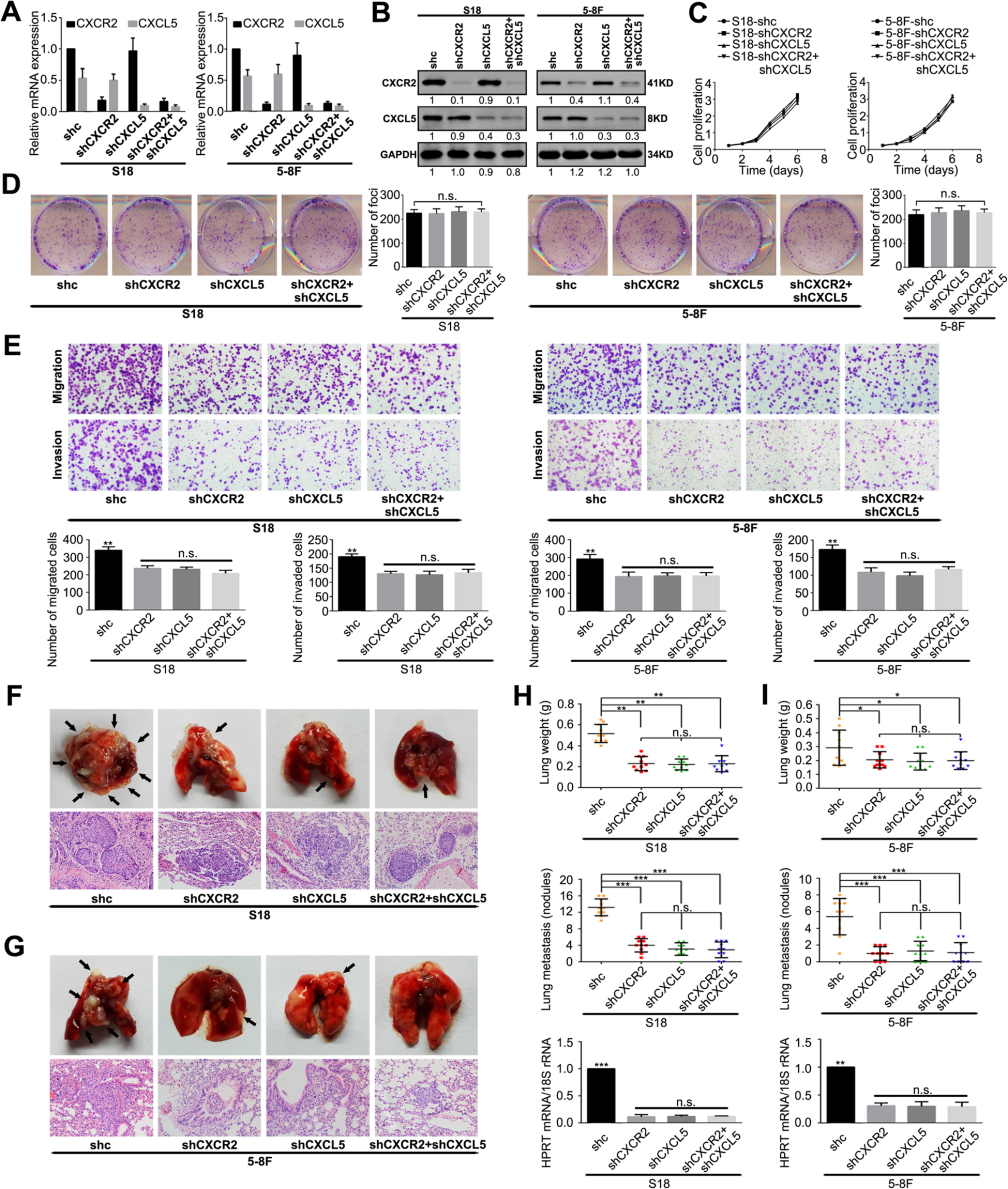
Silencing of CXCL5/CXCR2 axis inhibits migration and invasion in vitro and lung metastasis in vivo. **a** The relative mRNA levels of CXCL5/CXCR2 in the indicated stable cell lines were determined by qRT-PCR. The mean ± SD of triplicate samples are shown; *n* = 3. **b** The decreased expression of CXCL5 and CXCR2 in the CXCL5/CXCR2-silenced S18 and 5-8F cells compared with the scramble shRNA control cells were confirmed by western blotting. **c** The cell growth rates were compared between the CXCR2-, CXCL5-, CXCR2 + CXCL5- and scramble shRNA-transfected control cells using cell growth assays. **d** Representative images and summaries of the foci formation induced by cells transfected with CXCL5 and CXCR2 shRNA and scramble shRNA control cells. **e** The cell migration and invasion of the indicated stable cell lines were determined as described in the Materials and Methods. Representative images of the migrated and invaded cells are shown. **f**, **g** The indicated stable transfected cells were injected into the lateral tail veins of nude mice. The superior and inferior panels present the macroscopic appearance of metastatic lung tumours and H & E staining, respectively. The arrows indicate the metastatic nodules. Original magnification: 20 × objective. **h**, **i** The tumour weights and numbers of metastases per lung in the mice that were injected with the indicated stable cells, and the expression of human HPRT mRNA relative to mouse 18S rRNA in the lungs of the tumour-bearing nude mice are shown in H and I, respectively. There were 10 mice in each group. These experiments were repeated at least three times. Error bars, mean ± SD. *P < 0.05, **P < 0.01, ***P < 0.001

The S18 and 5-8F cell lines were transfected with CXCL5-specific and/or CXCR2-specific shRNAs to decrease the expression of CXCL5 and/or CXCR2. Nonspecific shRNA was used as a negative control (shc). We found that the mRNA and protein levels of CXCL5 and/or CXCR2 were significantly decreased after transfection with CXCL5-specific and/or CXCR2-specific shRNA (Fig. 3a and b). The results were consistent with those obtained using an ELISA to determine the levels of CXCL5 in the cell culture supernatants (Additional file 5: Figure S2). As expected, in both cell lines, CXCL5-silencing and/or CXCR2-silencing significantly attenuated the migration and invasion abilities without influencing cell proliferation (Fig. 3c-e).

We developed an in vivo lung metastasis model by injecting S18 or 5-8F and their derived cells into the lateral tail veins of nude mice for 7 weeks. The results indicated that the silencing of CXCL5 and/or CXCR2 could significantly decrease distant metastasis of NPC cells (Fig. 3f-i).

### Overexpression of the CXCL5/CXCR2 axis promotes the metastasis of NPC cells through the induction of the EMT

**Fig. 4:**
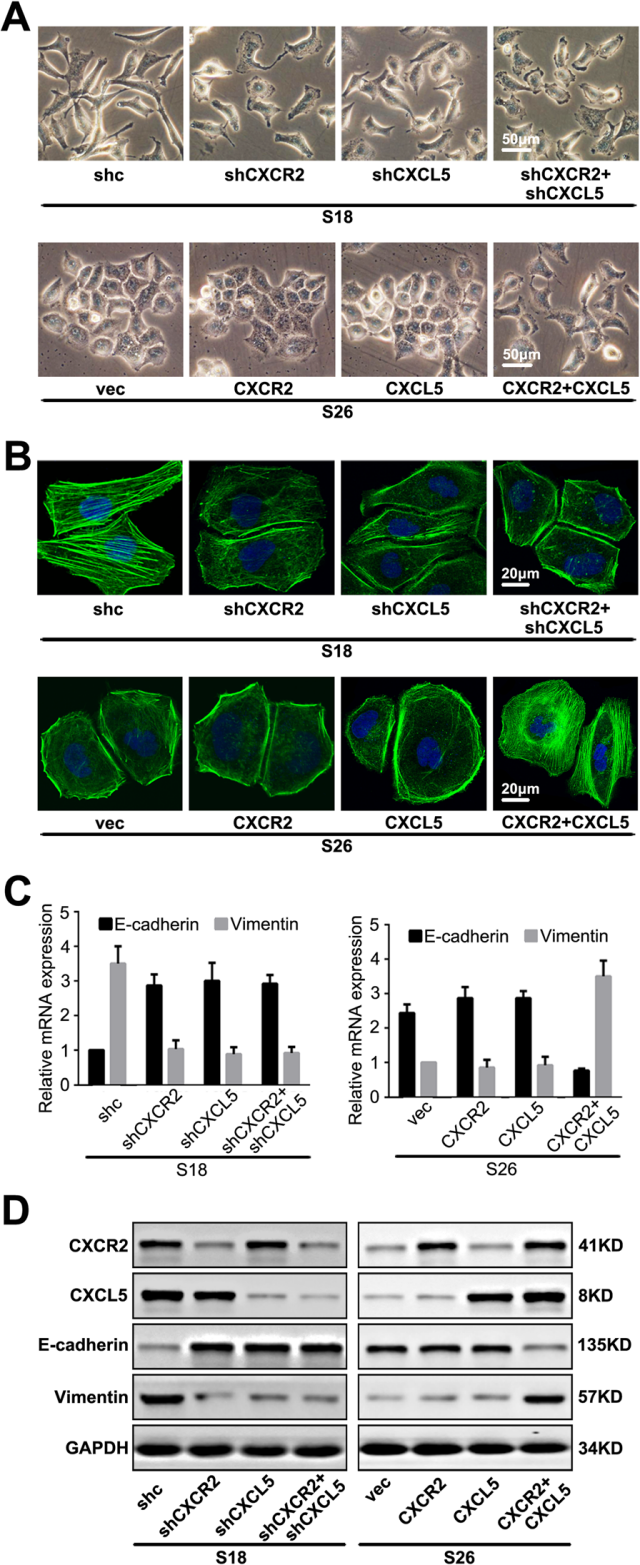
CXCL5/CXCR2 together induces EMT in NPC cells. **a** The cellular morphologies of the NPC cells with high or low CXCL5/CXCR2 expression. **b** The influence of the CXCL5/CXCR2 expression levels on cell morphology was confirmed by immunofluorescence assays using phalloidin (green) with DAPI counterstaining (blue). **c** The results of qRT-PCR and (**d**) western blot analyses revealed changes in the expression of EMT markers (E-cadherin and Vimentin) in the NPC cell lines. These experiments were repeated at least three times. Error bars, mean ± SD

As shown in Fig. 4a, the NPC cells that expressed high levels of only one of the proteins or neither protein presented with the typical cobblestone-like appearance of normal epithelium, whereas the S18-shc and S26-CXCR2-CXCL5 cells with high CXCL5/CXCR2 expression levels both took on spindle-like, fibroblastic morphologies. The influence of CXCL5/CXCR2 expression levels on cell morphology was further confirmed by immunofluorescent staining for phalloidin (Fig. 4b). Western blotting indicated that the S18-shc and S26-CXCR2-CXCL5 cells exhibited the typical EMT phenotype, including the downregulation of E-cadherin and the upregulation of Vimentin (Fig. 4c and d). Taken together, these data suggested that the overexpression of the CXCL5/CXCR2 axis could promote the EMT process.

### Snail is required for CXCL5/CXCR2 axis-triggered EMT and invasion capacity of NPC cells

**Fig. 5:**
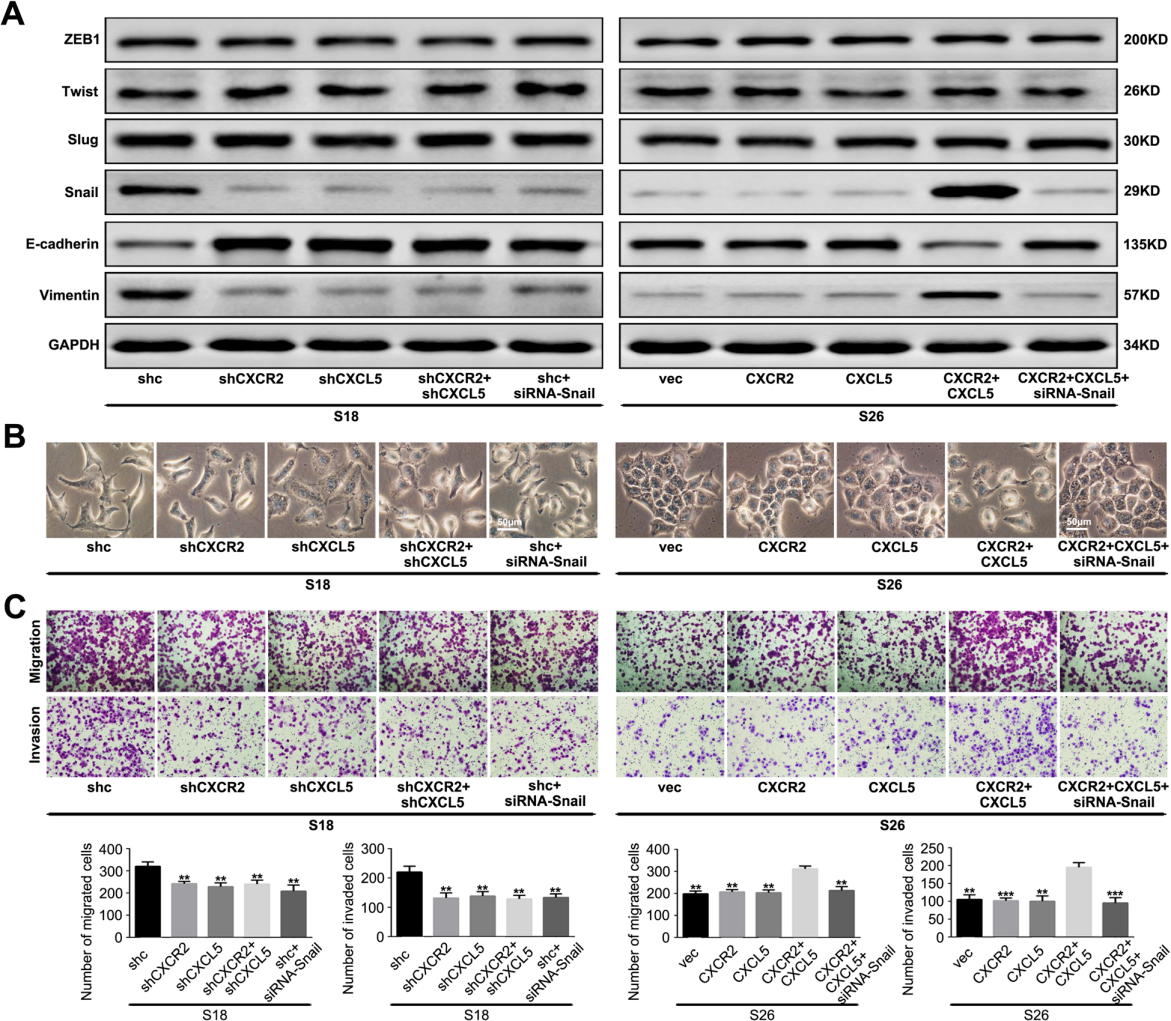
Snail is required for CXCL5/CXCR2 axis-triggered EMT. **a** The expression profiles of ZEB1, Twist, Slug and Snail in the stable transfected cells based on western blot analysis. The knockdown of Snail with small interfering RNA reversed the CXCL5/CXCR2 axis-triggered EMT phenotype in the NPC cells as revealed by a western blot analysis and (**b**) cellular morphology. **c** The knockdown of Snail abolished the invasive ability of the NPC cells that was induced by the CXCL5/CXCR2 axis. These experiments were repeated at least three times. Error bars, mean ± SD. **P < 0.01, ***P < 0.001

To further characterize the transcriptional factors involved in the EMT triggered by the CXCL5/CXCR2 axis, we examined the expression profiles of ZEB1, Twist, Slug and Snail in NPC cells. As shown in Fig. 5a, we found significantly increased levels of Snail in the CXCR2- and CXCL5-transfected S26 cells and decreased levels of Snail in the shRNA-CXCR2- and/or shRNA-CXCL5-treated S18 cells. However, we observed no significant changes in the ZEB1, Twist, or Slug levels. Next, after the introduction of siRNA specific for Snail into the S18-shc and S26-CXCR2-CXCL5 cells, we found that E-cadherin expression was enhanced, and Vimentin was downregulated (Fig. 5a). Additionally, the spindle-like, fibroblastic morphology appeared to be abolished, and the migration and invasion abilities of these cells were decreased compared with the control cells (Fig. 5b and c). These results indicated that the CXCL5/CXCR2 axis-induced EMT and invasive capacity of the NPC cells were dependent on the transcriptional factor Snail.

### The CXCL5/CXCR2 axis leads to the activation of GSK-3β by phosphorylation

**Fig. 6:**
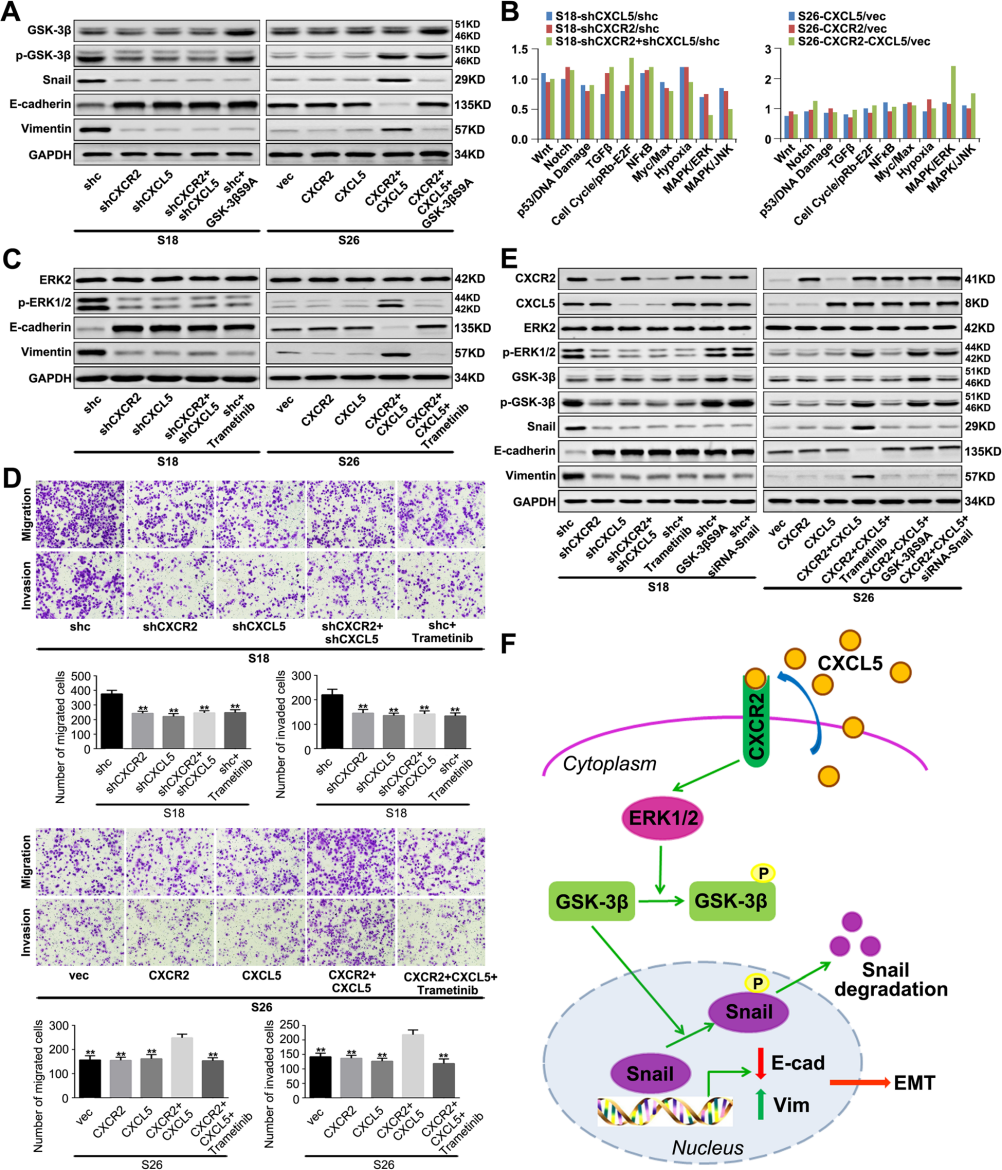
The CXCL5/CXCR2 axis contributes to the EMT of NPC cells by activating ERK/GSK-3β/Snail signalling. **a** GSK-3β participated in the CXCL5/CXCR2 axis-induced EMT of NPC cells. **b** Luciferase reporter assays were performed with the indicated stable transfected cells. The transcriptional activity of the ERK1/2 pathway was enhanced predominantly when both proteins in the CXCL5/CXCR2 axis were overexpressed. **c** Western blot analysis showed that CXCL5/CXCR2 together induces hyperactivity of ERK1/2 signalling, which is necessary for the EMT. **d** ERK1/2 signalling is necessary for the invasiveness of NPC cells that is induced by the CXCL5/CXCR2 axis. **e** The CXCL5/CXCR2 axis contributes to the EMT of NPC cells by activating ERK/GSK-3β/Snail signalling. **f** Schematic representation of the major molecular mechanism of the induction of the EMT in NPC cells by the CXCL5/CXCR2 axis. These experiments were repeated at least three times. Error bars, mean ± SD. **P < 0.01, ***P < 0.001

GSK-3β and nuclear factor-κB (NF-κB) have previously been demonstrated to mediate the stabilization of endogenous Snail [20, 41]. Therefore, we evaluated the levels of P65 NF-κB and GSK-3β and their phosphorylation statuses in NPC cells. Western blotting revealed no significant difference in the level of activated P65 between these cells (Additional file 6: Figure S3). However, the phosphorylation of GSK-3β was notably enhanced in the NPC cells with high expression levels of both CXCL5/CXCR2 together, and the expression of Snail changed in parallel with p-GSK-3β (Fig. 6a). These findings indicated that the effect of the CXCL5/CXCR2 axis on the stabilization of Snail in the NPC cells was dependent on the phosphorylation status of GSK-3β.

Then, a constitutively active mutant of GSK-3β (GSK-3βS9A) was introduced into S18-shc and S26-CXCR2-CXCL5 cells. The expected enhanced expression of GSK-3β and reduced expression of Snail are presented in Fig. 6a. An analysis of the expression of epithelial and mesenchymal markers in these cells revealed that E-cadherin expression was enhanced, whereas Vimentin was downregulated (Fig. 6a). These results indicated that the upregulation of p-GSK-3β promoted an EMT phenotypic change in the NPC cells that was triggered by the CXCL5/CXCR2 axis.

### High expression levels of both CXCL5/CXCR2 together results in increased activity of the ERK1/2 signalling pathway as measured by luciferase reporter assays

The luciferase reporter assays revealed that the transcriptional activity of the ERK1/2 pathway was significantly enhanced when both members of the CXCL5/CXCR2 axis were highly expressed (Fig. 6b). A western blot analysis revealed that the S18-shc and S26-CXCR2-CXCL5 cells exhibited higher levels of ERK phosphorylation than did the other stable cells. In contrast, neither the expression of ERK2 nor total ERK1/2 was altered regardless of the expression levels of CXCL5/CXCR2 (Fig. 6c and Additional file 7: Figure S4). Additionally, CXCL5 has been demonstrated to possess the ability to activate the PI3K-AKT signalling pathway in hepatocellular carcinomas [20] and colorectal cancer [25] cells via its receptor CXCR2. Therefore, we also evaluated the PI3K-AKT pathway in NPC cells. As illustrated in Additional file 8: Figure S5, the expression levels of CXCL5 and/or CXCR2 did not affect AKT phosphorylation in the S18 and S26 stable cell lines. These results demonstrated that CXCL5/CXCR2 triggered the activation of ERK by phosphorylation.

### CXCL5/CXCR2 together induces hyperactivity of ERK1/2 signalling, which is necessary for the EMT and invasiveness of NPC cells

Western blot analysis (Fig. 6c) indicated that trametinib (an ERK1/2 inhibitor) successfully reduced the phosphorylation levels of the corresponding proteins and led to an epithelial phenotype in the NPC cells. Additionally, another ERK inhibitor, U0126, proved the effect of the phosphorylation of activated ERK on GSK-3β (Additional file 9: Figure S6). Moreover, the invasive activities of these cells were inhibited by trametinib (Fig. 6d). To determine whether the migration inhibitory effect of trametinib on NPC cells was associated with cell death, flow cytometry analyses were performed on S18-shc and S26-CXCR2-CXCL5 cells that were treated with 50 nM trametinib (the same concentration used in the migration assay) for 24 h. As depicted in Additional file 10: Figure S7, trametinib did not induce apoptosis in NPC cells. These results suggested that ERK1/2 signalling is necessary for the EMT and the invasiveness of the NPC cells induced by the CXCL5/CXCR2 axis.

### The CXCL5/CXCR2 axis contributes to EMT by activating the ERK/GSK-3β/snail pathway in NPC cells

The results presented above suggest that the ERK/GSK-3β/Snail cascade is initiated by the CXCL5/CXCR2 axis. To further verify this suggestion, we compared the expression levels of these proteins in all of the experimental cells. As shown in Fig. 6e and Additional file 7: Figure S4, in the NPC cells that coexpressed high levels of CXCR2 and CXCL5 (i.e., S18-shc and S26-CXCR2-CXCL5 cells), the levels of p-ERK1/2, p-GSK-3β, and Snail were enhanced, and we observed the typical EMT phenotype, which included the downregulation of E-cadherin and the upregulation of Vimentin. When the S18-shc or S26-CXCR2-CXCL5 cells were treated with trametinib (an ERK1/2 inhibitor), the phosphorylation level of GSK-3β (Ser9) and the expression of Snail were reduced, and these reductions were accompanied by a reversal of the EMT phenotype, whereas the CXCR2 and CXCL5 expression was unchanged. After constitutively active GSK-3β (Ser9) plasmids were transfected into S18-shc or S26-CXCR2-CXCL5 cells, the expression of Snail was markedly reduced and this reduction was accompanied by a reversal of the EMT phenotype, but the CXCR2, CXCL5, and p-ERK1/2 expression levels remained unchanged. When siRNA interfered with the expression of Snail in S18-shc or S26-CXCR2-CXCL5 cells, we observed an upregulation of E-cadherin and a downregulation of Vimentin, but none of the aforementioned signalling molecules showed altered levels. These results further confirmed that the CXCL5/CXCR2 axis induces the EMT through the activation of the ERK/GSK-3β/Snail signalling pathway (Fig. 6f).

## Discussion

The CXCL5/CXCR2 signalling pathway plays a promoting role in some common human malignancies [18–20, 25, 29, 42], but the exact roles of the CXCL5/CXCR2 axis in NPC cell migration and invasion, as well as the underlying mechanisms, remain unclear.

In the present study, we found that CXCL5 and CXCR2 were upregulated in NPC tissues compared with those in non-tumour tissues from patients with chronic nasopharyngitis. High levels of CXCL5 were significantly associated with advanced nodal classification and progression, and high levels of CXCR2 were significantly correlated with advanced nodal classification and metastasis. Further investigation revealed that the expression levels of CXCL5 and CXCR2 were also significantly increased in the NPC cell lines compared with those in immortalized nasopharyngeal epithelial cell line NP69. The CXCL5/CXCR2 axis promoted lung metastasis in vivo and contributed to NPC cell migration and invasion via the ERK/GSK-3β/Snail pathway through the induction of the EMT in vitro.

CXCL5 can bind CXCR2 to mediate various cellular behaviours that include neutrophil recruitment and tumour cell migration and invasion [43]. Different cell types, such as immune cells, stromal cells, and astrocytes, are able to secrete CXCL5 [44–46]. Recent studies have reported that CXCL5 might promote migration and invasion in autocrine- and paracrine-dependent manners in glioma [42] and osteosarcoma [29] cells. However, in colorectal cancer, the effect of CXCL5 may likely depend on an autocrine signalling pathway [25]. In our study, CXCL5 was primarily secreted by tumour tissues rather than by the mesenchymal cells, and the NPC cell lines were capable of expressing CXCL5. These results suggest that CXCL5 promotes the migration and invasion of NPC cells in an autocrine manner.

In the present study, the expression levels of CXCL5 and CXCR2 were higher in the highly metastatic cell lines (S18 and 5-8F) than in the poorly metastatic cell lines (S26 and 6-10B). Only the overexpression of both CXCL5 and CXCR2 promoted the migration and invasion of the NPCs. The silencing of CXCL5 and/or CXCR2 decreased the migration and invasion of the NPCs. However, no differences in the migration and invasion assays were noted in the cells that expressed high levels of CXCR2 or CXCL5 alone or low levels of CXCL5/CXCR2 together. We postulate that the similar invasive and metastatic potentials of these cell lines may be explained as follows: in the poorly metastatic cell lines (i.e., S26 and 6-10B), after the overexpression of CXCR2, additional CXCL5 secreted by the NPC cells could combine with CXCR2. Nevertheless, the enhanced effect of this combination was too weak to induce functional changes (migration/invasion) because the basic level of CXCL5 was low in these cell lines. Moreover, a high level of CXCL5 expression alone could not enhance the migratory and invasive abilities because of the low level of CXCR2 in these cell lines. In contrast, in highly metastatic cell lines (i.e., S18 and 5-8F), we suppose that the silencing efficiencies of the RNA interference (RNAi) technique on the expression of CXCL5 or CXCR2 were very high. Therefore, the silencing of CXCL5 or CXCR2 alone resulted in dramatic functional changes, and there was no significant difference in the migration and invasion assay results between these two groups. Moreover, the in vitro functional changes are tightly consistent with morphologic and molecular changes related to the EMT in NPC cells. For example, mesenchymal markers were significantly upregulated in the NPC cell lines that highly expressed both CXCR2 and CXCL5, whereas typical epithelial markers were markedly decreased. However, the cells that only expressed high levels of one of the proteins and those that expressed neither of the proteins did not present these changes. Our findings are consistent with those of Zhou’s study [20], which demonstrated that the CXCL5/CXCR2 axis promotes invasion and triggers the EMT in HCC cells.

The ERK pathway has significant roles in cell proliferation, cell survival and metastasis [47]. Aberrant activation of the ERK pathway may be involved in the progression of a variety of tumours, such as nasopharyngeal carcinomas [48], breast cancer [49], and pancreatic adenocarcinomas [50]. In Zhou’s study, CXCL5 was demonstrated to have the ability to activate the PI3K-Akt and ERK1/2 signalling pathways in HCC cells via its receptor CXCR2 [51]. These authors further confirmed that PI3K/Akt signalling, but not ERK1/2 signalling, was necessary for the EMT and the invasiveness of HCC cells induced by the CXCR2/CXCL5 axis [20]. A previous study by Gao et al. found that the CXCL5/CXCR2 axis promotes bladder cancer cell migration and invasion by activating the PI3K/AKT-induced upregulation of MMP2/MMP9 [19]. Zhao et al. [25] demonstrated that the CXCL5/CXCR2 axis induced the EMT in CRC cells via the ERK/Elk-1/Snail pathway while promoting CRC cell invasion through the AKT/GSK3β/β-catenin/MMP7 pathway. In our study, the luciferase reporter assays revealed that the transcriptional activity of the ERK1/2 pathway was enhanced significantly when the CXCL5/CXCR2 axis was overexpressed (Fig. 6b). Western blot analysis further demonstrated that CXCL5/CXCR2 axis triggered the activation of ERK, but not AKT, by phosphorylation. Therefore, our research suggested that the CXCL5/CXCR2 axis contributes to migration and invasion by activating the ERK1/2 signalling pathway in NPC cells, which implies that this axis might participate in extensive biological activities.

Snail is a zinc-finger transcription factor that is known as an essential player in the aggressive phenotype of the EMT [52]. Snail binds to the E-boxes of the human E-cadherin promoter and represses E-cadherin expression. We observed marked increases in the protein level of Snail and concomitant decreases in the expression level of E-cadherin in the CXCL5- and CXCR2-overexpressing cells. In many cells, GSK-3β is constitutively active and can bind and phosphorylate Snail to facilitate its degradation [53]. In contrast, inactivation of GSK-3β promotes Snail stabilization, nuclear translocation, and subsequent EMT induction [53]. In the current study, western blotting revealed that the CXCL5/CXCR2 axis increased the phosphorylation of GSK-3β (Ser9) and stimulated the nuclear translocation of Snail in NPC cells. Conversely, we observed reduced expression of Snail in cells after transfection with GSK-3βS9A. Our data suggested that the CXCL5/CXCR2 axis enhanced the phosphorylation of GSK-3β at serine 9 in the active site, which led to its disassociation from Snail and thereby facilitated the nuclear translocation of Snail and the EMT.

Based on our findings, we propose a mechanism for the CXCL5/CXCR2 axis-induced EMT in NPC cells, as shown in Fig. 6f. The overexpression of the CXCL5/CXCR2 axis leads to the activation of the ERK1/2 signalling pathway. Activated ERK1/2 causes the phosphorylation and resultant inactivation of GSK-3β, which results in the dissociation of GSK-3β and Snail. The unbound Snail then migrates to the nucleus and functions as a repressor of E-cadherin, which eventually leads to a mesenchymal-like phenotypic alteration of NPC cells.

The concomitant expression of CXCL5 and CXCR2 makes a substantial contribution to the tumour progression of NPCs. Therefore, the development of CXCR2 antagonists may be of use in antitumour therapy. In previous studies, researchers have demonstrated that treatment with a CXCR2 antagonist (SB225002) inhibits the tumour cell invasive properties of lung adenocarcinomas [23] and suppresses the tumour growth of intrahepatic cholangiocellular carcinomas [54]. Additionally, Varney’s study reported that orally active CXCR2/1 antagonists (SCH-527123 and SCH-479833) inhibit the human colon cancer liver metastasis mediated in a manner that is mediated by decreased tumour vascularity and increased malignant cell apoptosis [55]. Furthermore, SCH-527123 has been proven to be capable of suppressing CXCR2-mediated signal transduction through decreased phosphorylation of the NF-kB/mitogen-activated protein kinase (MAPK)/AKT pathway, which leads to decreased cell migration and invasion and increased apoptosis in colorectal cancer cell lines [56]. The effectiveness and mechanism of CXCR2 antagonists in the inhibition of NPC growth and metastasis remain unclear, and this issue is the key point of our future study.

## Conclusions

In summary, we found that the CXCL5/CXCR2 axis promotes NPC cell migration, invasion, and metastasis via the activation of the ERK/GSK-3β/Snail signalling pathway and the induction of the EMT, which may lead to the identification of new therapeutic targets for the distant metastasis of NPC.

## Additional files

Additional file 1: **Table S1.** Median and range of the IRS for each antibody. (DOCX 13 kb)

Additional file 2: **Table S2.** Primers and vshRNAs/siRNAs used in the study. (DOCX 14 kb)

Additional file 3: **Table S3.** Primary antibodies used for the western blot, immunohistochemistry and immunofluorescence analyses. (DOCX 15 kb)

Additional file 4: **Figure S1.** The CXCL5 levels in the conditioned media (CM) of the S26 (A) and 6-10B (B) stable cell lines were detected by ELISA. (TIFF 134 kb)

Additional file 5: **Figure S2.** The CXCL5 levels in the conditioned media (CM) of the S18 (A) and 5-8F (B) stable cell lines were detected by ELISA. (TIFF 178 kb)

Additional file 6: **Figure S3.** Western blotting showed that the expression of CXCL5 and/or CXCR2 did not affect P65 phosphorylation in the S18 and S26 stable cell lines. (TIFF 253 kb)

Additional file 7: **Figure S4.** Western blotting showed that there is no difference in the expression of ERK2 or total ERK1/2 after different treatment in the S18 and S26 stable cell lines. (TIFF 237 kb)

Additional file 8: **Figure S5.** Western blotting showed that the expression of CXCL5 and/or CXCR2 did not affect AKT phosphorylation in the S18 and S26 stable cell lines. (TIFF 241 kb)

Additional file 9: **Figure S6.** Western blotting showed that both ERK inhibitors (i.e., trametinib and U0126) reduced the phosphorylation levels of the corresponding proteins and led to an epithelial phenotype in the NPC cells. (TIFF 509 kb)

Additional file 10: **Figure S7.** Flow cytometry analyses were performed on the S18-shc (superior panel) and S26-CXCR2-CXCL5 (inferior panel) cells that were treated with 50 nM of trametinib for 24 h. As depicted in Fig. S7, trametinib did not induce apoptosis in the NPC cells. (TIFF 485 kb)

## Abbreviations

BCa: Bladder cancer
CRC: Colorectal cancer
DMEM: Dulbecco’s modified eagle’s medium
DMFS: Distant metastasis-free survival
ELISA: Enzyme-linked immunosorbent assay
EMT: Epithelial-mesenchymal transition
ERK: Extracellular signal-regulated kinase
GSK-3β: Glycogen synthase kinase-3 beta
H & E: Haematoxylin and eosin
HCC: Hepatocellular carcinoma
hHPRT: Human hypoxanthine-guanine-phosphoribosyl transferase
IHC: Immunohistochemical staining
NPC: Nasopharyngeal carcinoma
OS: Overall survival
PBS: Phosphate buffered saline
PI: Propidium idodide
qRT-PCR: Quantitative real-time polymerase chain reaction
shRNA: Short hairpin RNA
siRNA: Small interfering RNA
