# Supplementary material for: The CXCL5/CXCR2 axis contributes to the epithelial-mesenchymal transition of nasopharyngeal carcinoma cells by activating ERK/GSK-3β/snail signalling
Source: J Exp Clin Cancer Res. 2018 Apr 17;37:85. doi: 10.1186/s13046-018-0722-6 (PMC5905166; doi:10.1186/s13046-018-0722-6)
Supplement: Supplementary file 1 — Table S1. Median and range of the IRS for each antibody. (DOCX 13 kb) [file 13046_2018_722_MOESM1_ESM.docx]

**Supplementary Table 1. Median and range of the IRS for each antibody**

|  | | CXCL5 | | CXCR2 | |
| --- | --- | --- | --- | --- | --- |
| Group | | Low | High | Low | High |
| Median | | 2 | 4 | 2 | 4 |
| Range | | 0-3 | 4-6 | 0-3 | 4-6 |
| Total | Median | 3 | | 3 | |
|  | Range | 0-6 | | 0-6 | |
