# Supplementary material for: The CXCL5/CXCR2 axis contributes to the epithelial-mesenchymal transition of nasopharyngeal carcinoma cells by activating ERK/GSK-3β/snail signalling
Source: J Exp Clin Cancer Res. 2018 Apr 17;37:85. doi: 10.1186/s13046-018-0722-6 (PMC5905166; doi:10.1186/s13046-018-0722-6)
Supplement: Supplementary file 2 — Table S2. Primers and vshRNAs/siRNAs used in the study. (DOCX 14 kb) [file 13046_2018_722_MOESM2_ESM.docx]

**Supplementary Table 2. Primers and vshRNAs/siRNAs used in the study**

| **Primers /vshRNAs/siRNAs sequences (5’------3’)** |
| --- |
| **Primers for qRT-PCR:**  CXCR2    sense 5’-CCTGTCTTACTTTTCCGAAGGAC-3’    antisense‌‌‌‍ 5’-TTGCTGTATTGTTGCCCATGT-3’  CXCL5 sense 5’-AGCTGCGTTGCGTTTGTTTAC-3’    antisense 5’-TGGCGAACACTTGCAGATTAC-3’  E-cadherin   sense 5’-AATAGTGCCTAAAGTGCTGC -3’  antisense  5’-AGACCCACCTCAATCATCCT-3’  Vimentin    sense 5’-GACGCCATCAACACCGAGTT-3’    antisense 5’-CTTTGTCGTTGGTTAGCTGGT-3’  hHPRT    sense 5’-TTCCTTGGTCAGGCAGTATAATCC-3’    antisense 5’-AGTCTGGCTTATATCCAACACTTCG-3’  18sRNA    sense 5’-TTCCTTGGTCAGGCAGTATAATCC-3’    antisense 5’-AGTCTGGCTTATATCCAACACTTCG-3  GAPDH     sense 5’-ACAGTCAGCCGCATCTTCTT-3’    antisense 5’-GACAAGCTTCCCGTTCTCAG-3’  **shRNAs and siRNAs used in the study**  CXCR2:  shRNA#1   GGACTCCTCAAGATTCTAGCT  shRNA#2 GCTATACATGGCTTGATCAGC  shRNA#3 GCTATGAGGACATGGGCAACA  shRNA#4 CTATAGTGGCATCCTGCTACT  CXCL5:  shRNA#1    GCTGCGTTGCGTTTGTTTACA  shRNA#2 GGAAATACACTGTTATCTTCA  shRNA#3 GCTAATATCTTATCTTCCTAT  shRNA#4 GTGCTCCGGATCCTCCAATCT  Snail:  siRNA#1 AATCGGAAGCCTAACTACA  siRNA#2 GCTGCAGGACTCTAATCCA  siRNA#3 GAATGTCCCTGCTCCACAA |
