# Supplementary material for: The CXCL5/CXCR2 axis contributes to the epithelial-mesenchymal transition of nasopharyngeal carcinoma cells by activating ERK/GSK-3β/snail signalling
Source: J Exp Clin Cancer Res. 2018 Apr 17;37:85. doi: 10.1186/s13046-018-0722-6 (PMC5905166; doi:10.1186/s13046-018-0722-6)
Supplement: Supplementary file 3 — Table S3. Primary antibodies used for the western blot, immunohistochemistry and immunofluorescence analyses. (DOCX 15 kb) [file 13046_2018_722_MOESM3_ESM.docx]

**Supplementary Table 3. Primary antibodies used for WB, IHC and IF**

| Protein | Concentration  for WB | Concentration  for IHC | Concentration  for IF | Specificity | Company |
| --- | --- | --- | --- | --- | --- |
| CXCR2 | 1:500 | 1:200 | / | Rabbit Polyclonal | Abcam |
| CXCL5 | 1:500 | 1:100 | / | Mouse Monoclonal | R&D Systems |
| ERK2 | 1:1000 | / | / | Rabbit Monoclonal | Proteintech |
| p-ERK1/2 | 1:1000 | / | / | Rabbit Monoclonal | Cell Signaling Technology |
| Total ERK1/2 | 1:1000 | / | / | Rabbit Monoclonal | Cell Signaling Technology |
| GSK-3β | 1:1000 | / | / | Rabbit Monoclonal | Cell Signaling Technology |
| p-GSK-3β | 1:1000 | / | / | Rabbit Monoclonal | Cell Signaling Technology |
| Snail | 1:1000 | / | / | Rabbit Monoclonal | Cell Signaling Technology |
| Slug | 1: 1000 | / | / | Rabbit Monoclonal | Cell Signaling Technology |
| Twist | 1:1000 | / | / | Mouse Monoclonal | Cell Signaling Technology |
| ZEB1 | 1: 1000 | / | / | Rabbit Monoclonal | Cell Signaling Technology |
| E-cadherin | 1: 1000 | / | / | Rabbit Monoclonal | Cell Signaling Technology |
| Vimentin | 1: 1000 | / | / | Rabbit Monoclonal | Cell Signaling Technology |
| Phalloidin | / | / | 1:100 | Mouse Monoclonal | Cell Signaling Technology |
| GAPDH | 1:1000 | / | / | Rabbit Monoclonal | Cell Signaling Technology |

Abbreviations: WB, western blot; IHC, immunohistochemistry; IF, immunofluorescence.
