# Supplementary figures and images for: The CXCL5/CXCR2 axis contributes to the epithelial-mesenchymal transition of nasopharyngeal carcinoma cells by activating ERK/GSK-3β/snail signalling
Source: J Exp Clin Cancer Res. 2018 Apr 17;37:85. doi: 10.1186/s13046-018-0722-6 (PMC5905166; doi:10.1186/s13046-018-0722-6)

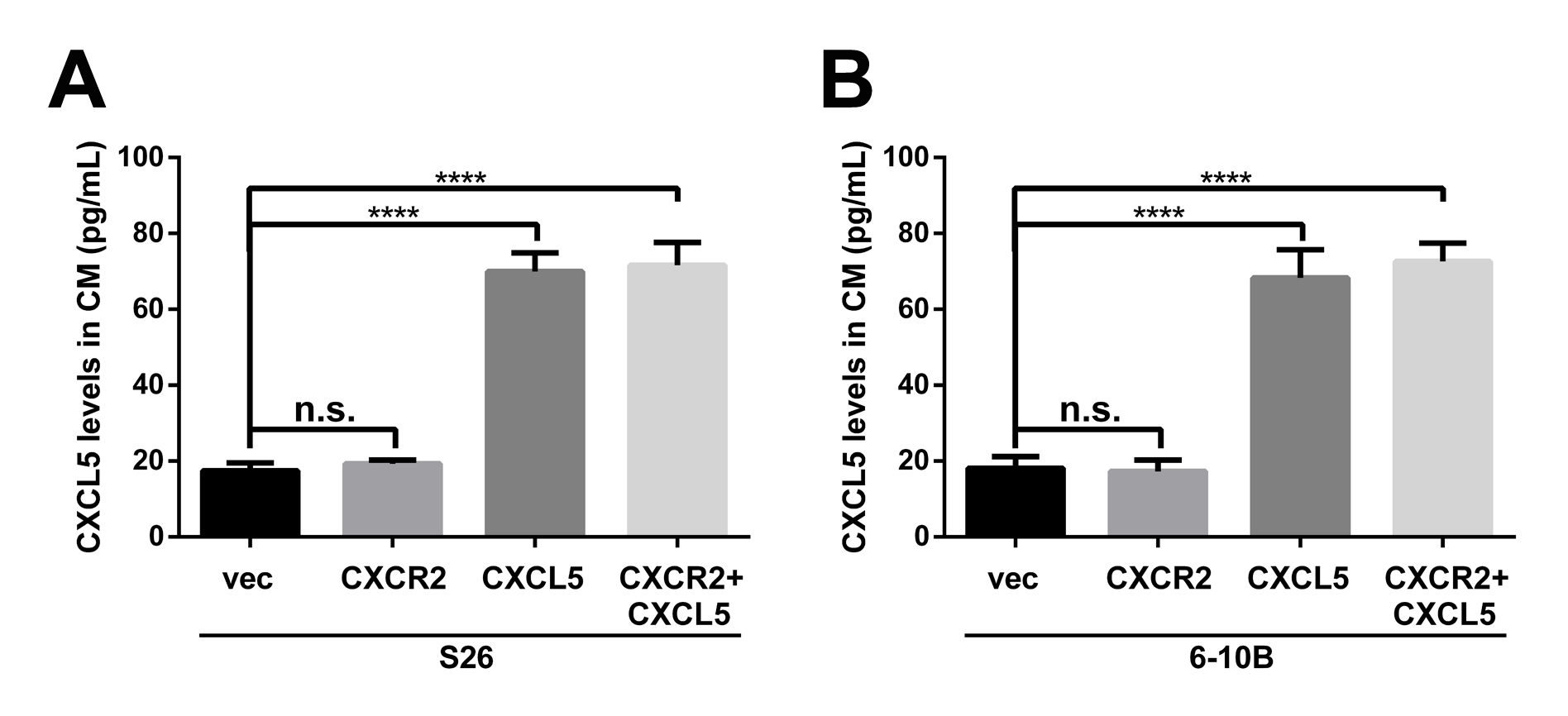

Supplement: Supplementary file 4 — Figure S1. The CXCL5 levels in the conditioned media (CM) of the S26 (A) and 6-10B (B) stable cell lines were detected by ELISA. (TIFF 134 kb) [file 13046_2018_722_MOESM4_ESM.tif]

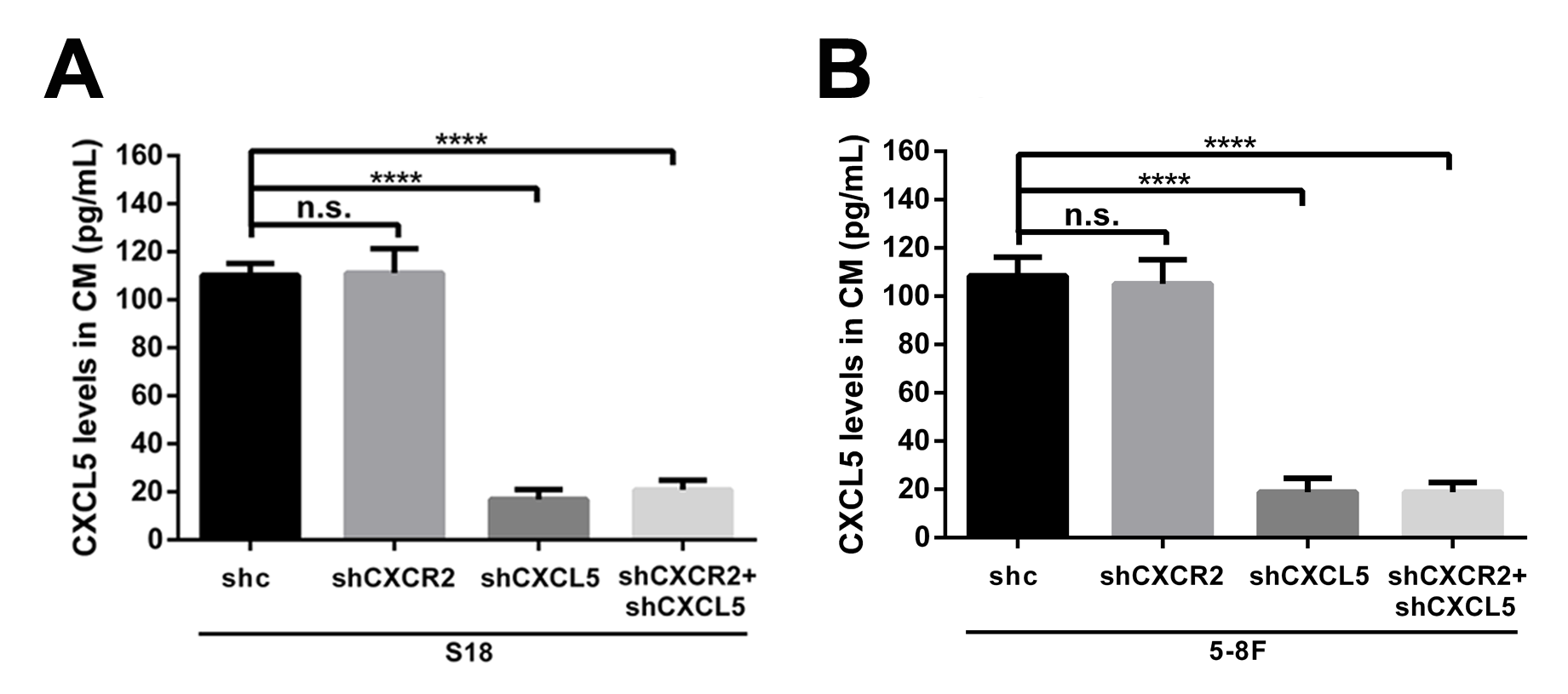

Supplement: Supplementary file 5 — Figure S2. The CXCL5 levels in the conditioned media (CM) of the S18 (A) and 5-8F (B) stable cell lines were detected by ELISA. (TIFF 178 kb) [file 13046_2018_722_MOESM5_ESM.tif]

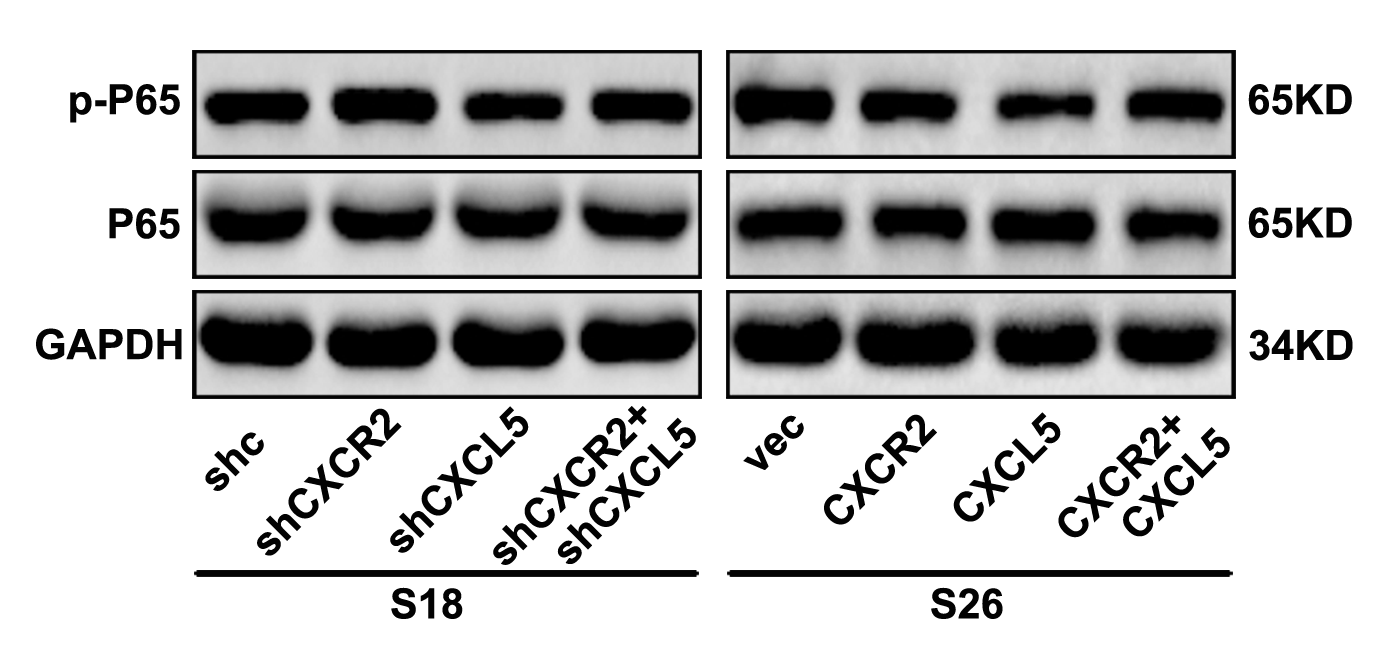

Supplement: Supplementary file 6 — Figure S3. Western blotting showed that the expression of CXCL5 and/or CXCR2 did not affect P65 phosphorylation in the S18 and S26 stable cell lines. (TIFF 253 kb) [file 13046_2018_722_MOESM6_ESM.tif]

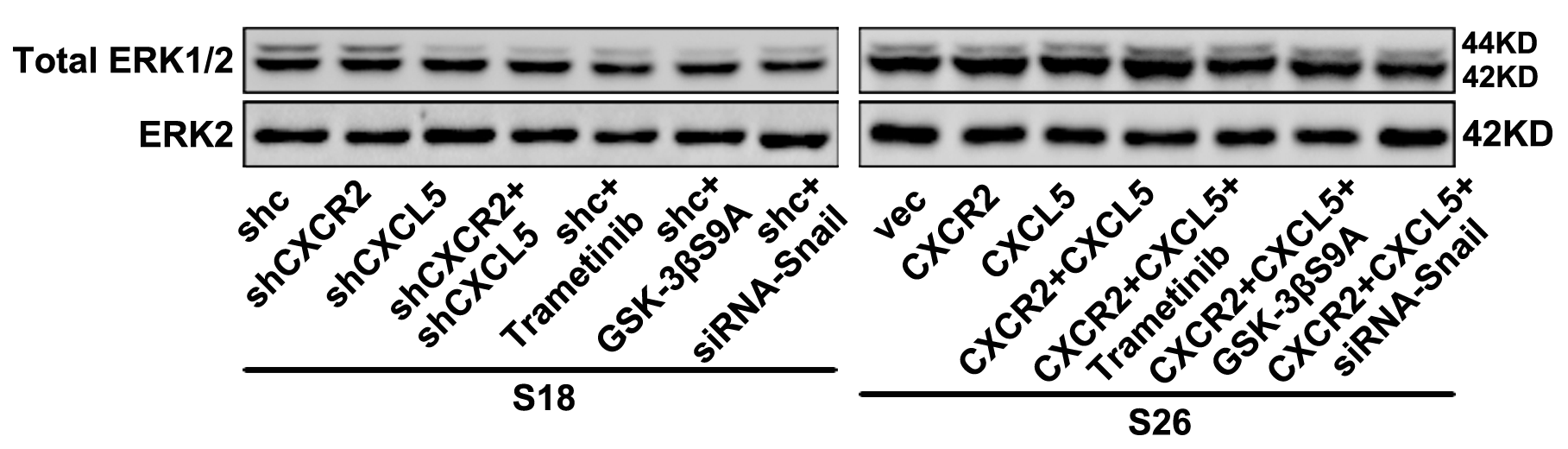

Supplement: Supplementary file 7 — Figure S4. Western blotting showed that there is no difference in the expression of ERK2 or total ERK1/2 after different treatment in the S18 and S26 stable cell lines. (TIFF 237 kb) [file 13046_2018_722_MOESM7_ESM.tif]

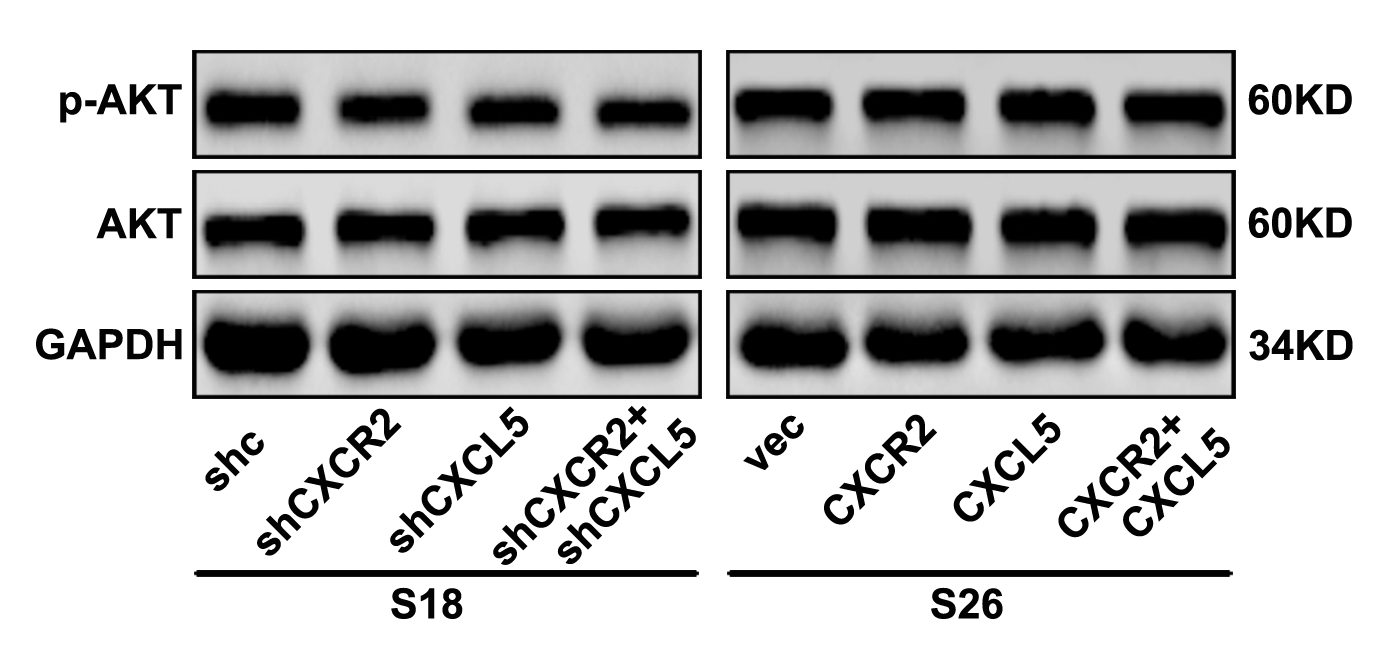

Supplement: Supplementary file 8 — Figure S5. Western blotting showed that the expression of CXCL5 and/or CXCR2 did not affect AKT phosphorylation in the S18 and S26 stable cell lines. (TIFF 241 kb) [file 13046_2018_722_MOESM8_ESM.tif]

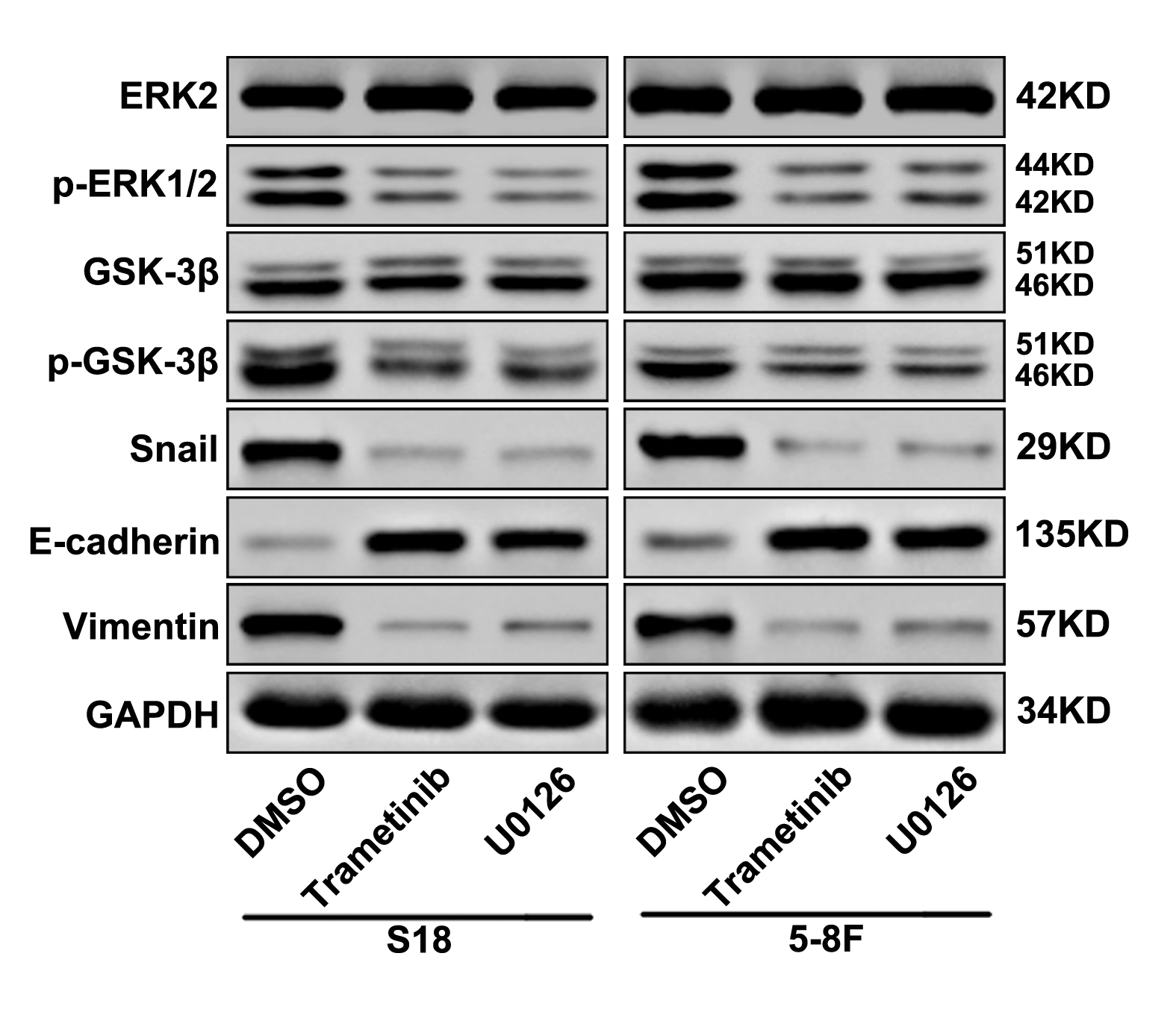

Supplement: Supplementary file 9 — Figure S6. Western blotting showed that both ERK inhibitors (i.e., trametinib and U0126) reduced the phosphorylation levels of the corresponding proteins and led to an epithelial phenotype in the NPC cells. (TIFF 509 kb) [file 13046_2018_722_MOESM9_ESM.tif]

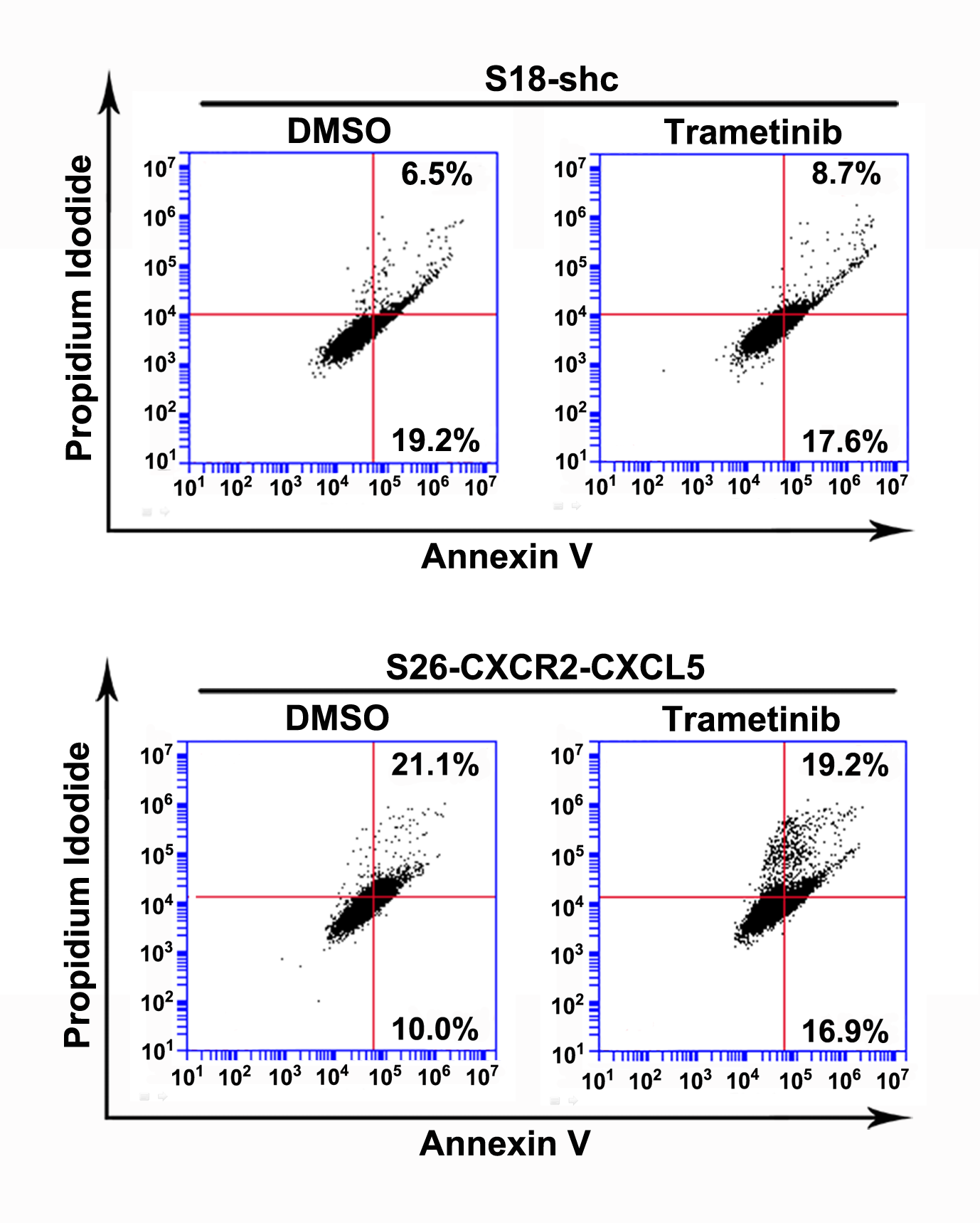

Supplement: Supplementary file 10 — Figure S7. Flow cytometry analyses were performed on the S18-shc (superior panel) and S26-CXCR2-CXCL5 (inferior panel) cells that were treated with 50 nM of trametinib for 24 h. As depicted in Fig. S7, trametinib did not induce apoptosis in the NPC cells. (TIFF 485 kb) [file 13046_2018_722_MOESM10_ESM.tif]
